# Commentary: The C9orf72 Repeat Expansion Disrupts Nucleocytoplasmic Transport

**DOI:** 10.3389/fneur.2016.00051

**Published:** 2016-03-31

**Authors:** Tudor Munteanu, Tim Lynch

**Affiliations:** ^1^Dublin Neurological Institute at Mater Misericordiae Hospital, Dublin, Ireland

**Keywords:** ALS, neurodegeneration, pathophysiology, nuclear pore complex proteins, RanGAP1

Amyotrophic lateral sclerosis (ALS) is a neurodegenerative disorder that affects the upper and lower motor neurons. It has a focal onset but gradually spreads, leading to disability and, eventually, death. About 5–10% of ALS is inherited, usually following a dominant pattern. Pathological analysis revealed that motor neuron degeneration and death in the familial and sporadic forms is closely connected to protein aggregation and deposition, abnormal level and function of RNA molecules, abnormal neuronal cytoarchitecture, and non-neuronal cell death (1).

The GGGGCC hexanucleotide expansion (G_4_C_2_ HRE) in chromosome 9 open reading frame 72 (C9orf72) gene is found in 40% of the familial ALS cases. It is also found in other neurodegenerative disorders, including frontotemporal dementias (FTDs) (2) and atypical parkinsonian syndromes (3)_._

The molecular mechanisms of C9orf72 ALS neurodegeneration are currently a subject of controversy. Two main pathophysiological models have been hypothesized with experimental evidence supporting both (4). The first is transcription of the HRE segments of the abnormal gene into abnormal RNA strands, which assemble into G-quadruplex structures, which directly interact with proteins and alter their function. The second is translation of this abnormal RNA into dipeptide repeat proteins (DPRs), which in turn can adversely influence cell function. Both of the above described mechanisms have the potential to influence the nuclear pore function by altering its protein complexes.

Zhang et al. have recently investigated the complex mechanisms that lead to impairment of the normal trafficking through the nuclear pore complex (NPC) associated with G_4_C_2_ HRE (5). They demonstrated that overexpression of RanGAP gene (which encodes a NPC protein – RanGAP1) rescued certain phenotypic traits associated with G_4_C_2_ HRE-mediated neurodegeneration by using (G_4_C_2_)_30_ *Drosophila* models. Different phenotypes were obtained by expressing the abnormal gene at different stages of fly development. If expressed in 1-day-old flies, the HRE sequence caused progressive defects in the photoreceptor organization, suggesting age-dependent neurodegeneration. Locomotor defects were noted in 15-day-old (G_4_C_2_)_30_ flies pointing toward motor neuron pathology. Both of these phenotypes were rescued either by RanGAP overexpression or by using a heterozygous RanGAP gain-of-function mutation. Conversely, photoreceptor degeneration was accelerated by RanGAP knockdown using RNA interference. When the (G_4_C_2_)_30_ HRE was expressed in larval motor neurons, it caused severe neuromuscular junction defects that were not rescued by RanGAP overexpression. These results suggested that RanGAP overactivity suppressed HRE-mediated neurodegeneration in adult *Drosophila*, but HRE-mediated neurotoxicity in the larval stage is RanGAP independent.

The authors also studied the RanGAP/G_4_C_2_ RNA interaction in induced pluripotent stem cell (iPSC) neurons, derived from multiple C9orf72 ALS patients and noted that RanGAP G_4_C_2_ RNA can colocalize in these cells. This interaction seems to lead to RanGAP loss-of-function, which was shown by a disrupted Ran nucleocytoplasmic gradient in cells expressing G_4_C_2_ HRE. HRE toxicity also appeared to be modulated by nucleocytoplasmic transport, and the data suggested that genetically enhancing nuclear import and/or inhibiting nuclear export can suppress G_4_C_2_-repeat-mediated degeneration in both *Drosophila* and human cells.

In order to prove that abnormal G_4_C_2_ HRE RNA is involved in the neurodegenerative pathophysiological pathway, the investigators treated C9 ALS iPSC neurons with antisense oligonucleotides targeting these molecules. The treatment rescued the disrupted Ran gradient, suggesting that the abnormal RNA may be responsible for the nucleocytoplasmic transport deficits. Neurodegenerative phenotype suppression also occurred using a nuclear export inhibitor, suggesting that nuclear export inhibition may compensate for disrupted nuclear import.

In summary, G_4_C_2_ HRE disrupts nucleocytoplasmic transport in *Drosophila* models and in human cells. RanGAP seems to be a key link in the pathophysiological chain, but other nuclear pore proteins may also be involved. Nuclear pore transport was previously implicated in both ALS and FTD pathophysiology, and data from this study suggest that the RNA fragments cause nucleocytoplasmic trafficking defects by direct interaction with NPC proteins. However, other authors argued that DPRs can cause a reversible dysfunction of the NPC in the absence of abnormal C9orf72 RNA (4). These mechanisms can be complementary, and further work is needed to elucidate a potential link between them. The results presented suggest that nuclear pore dysfunction is key to neurodegeneration in C9orf72 ALS. The abnormal phenotype is reversible by modifying the RanGAP gene, suggesting a potential therapeutic approach.

## Author Contributions

TM – summarized the original article, selecting important points for presentation, and wrote the manuscript. TL – manuscript review.

## Conflict of Interest Statement

The authors declare that the research was conducted in the absence of any commercial or financial relationships that could be construed as a potential conflict of interest.
